# Filtering of artificial chimeric reads generated by ligation preparation method of nanopore sequencing

**DOI:** 10.1016/j.isci.2026.115695

**Published:** 2026-04-14

**Authors:** Zihan Xie, Jiarong Zhang, Tingting Yang, Xiaochen Bo, Zhiguo Fu, Fengqin Yang, Fuqiang Ye, Ming Ni

**Affiliations:** 1 Academy of Military Medical Sciences, Beijing 100850, China; 2 College of Life Science and Technology, Beijing University of Chemical Technology, Beijing 100029, China; 3 School of Forensic Medicine, Shanxi Medical University, Jinzhong 030600, China; 4 School of Information Science and Technology, Northeast Normal University, Changchun 130117, People's Republic of China; 5 Huadong Research Institute for Medicine and Biotechniques, Nanjing 210002, People's Republic of China

**Keywords:** Biological sciences, Bioinformatics, Sequence analysis, Methodology in biological sciences, Artificial intelligence, Machine learning

## Abstract

Nanopore sequencing provides long and ultra-long reads that are valuable for structural variation (SV) detection and genome assembly. However, false-positive chimeric reads can arise and interfere with somatic SV calling. Here, we show that ligation-based library preparation generates false-positive chimeric reads, particularly inverted repeats, in both microbial and human DNA standards. The proportion of inverted repeats ranged from 0.18% to 7.33%, exceeding those observed in rapid and modified rapid preparations. Analysis of raw electrical signals revealed a characteristic smoothed segment at junction sites in nearly half of these chimeric reads. Based on these features, we developed a ResNet-based deep learning classifier to identify false-positive chimeric reads. The model achieved high accuracy in both human and microbial datasets and substantially reduced SV calling errors after filtering. These results demonstrate that library preparation-induced chimeric reads can be effectively detected and mitigated, improving the reliability of nanopore-based SV analysis.

## Highlights

• Nanopore sequencing produces FP chimeric reads affecting SV detection
• Ligation prep yields higher artificial chimeric rates than rapid prep
• FP chimeras show a smoothed signal at breakpoint junctions
• A ResNet model detects and filters FP chimeric reads accurately

## Graphical abstract

**Figure:**
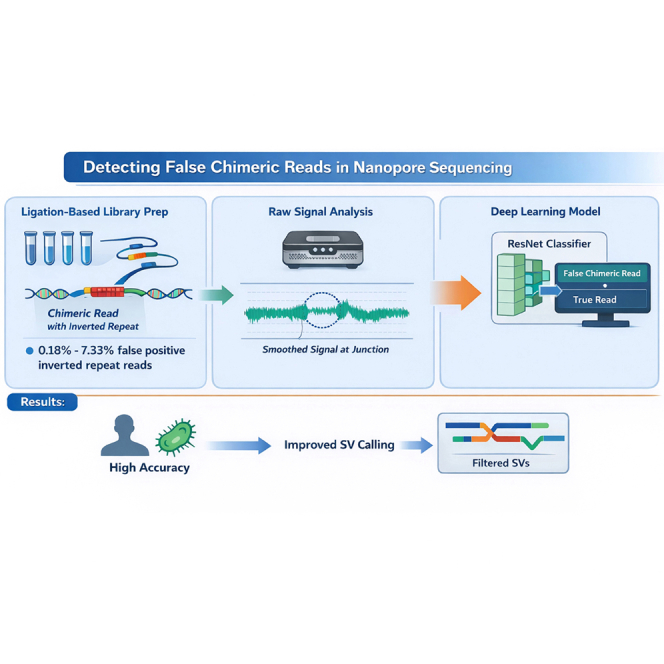

## Introduction

Chimeric reads, also referred to as split-read alignments,^1^ are DNA molecules composed of two or more distinct DNA sequence fragments, typically formed through recombination events involving different genomic regions. These DNA fragments can fuse to create a new DNA molecule with a unique sequence. Chimeras often represent structural variations (SVs) in genomes, where DNA fragments from the same or different chromosomes are mistakenly linked. When this incorrect linkage occurs specifically between the coding regions of two distinct genes, it is referred to as a fusion gene.^2^ Fusion genes are critical in the development of various cancers, such as sarcomas and epithelial-origin tumors.^3^^,^^4^ The identification of chimeric structures and fusion genes aids in detecting pathogenic mutations that may lead to disease and provides valuable insights for diagnosis and treatment.^5^^,^^6^

Advancements in sequencing technologies play a significant role in discovering gene fusions and chimeric phenomena. Next-generation sequencing (NGS) technology, widely used today, offers tremendous advantages over traditional Sanger sequencing, including speed, high throughput, and low cost.^7^ However, its short read lengths pose challenges in accurately identifying chimeric sequences and gene fusions. Additionally, NGS is affected by artifact chimeric reads, with reported rates ranging from 5.93% to 6.62%.^8^^,^^9^ In this study, artifact chimeric reads are defined as false positive (FP) chimeric reads to distinguish them from true structural variants (SVs). These FP chimeric reads typically result from amplification errors.^10^ The advent of third-generation sequencing (TGS) technologies has addressed some of these limitations by offering long reads, real-time analysis, and portability.^11^^,^^12^ The extended read lengths of TGS allow for more accurate alignment to reference genomes, significantly improving the detection of chimeric sequences.

Despite these advancements, TGS still encounters FP chimeric sequences. Amplification-based methods in TGS, such as PacBio sequencing, result in FP chimeric sequences in 42%–78% of cases.^13^ Even datasets generated by TGS without amplification may still produce FP chimeric reads.^14^^,^^15^ The generation of FP chimeric reads can be attributed to two main factors: (1) mis-priming during library preparation or amplification and (2) sequencing errors. Sequencing errors occur when two separate DNA fragments pass through a pore too quickly, causing the sequencing software to incorrectly interpret them as a single read. As a result, FP chimeric reads can arise with any library preparation method.

To address the impact of FP chimeric sequences on bioinformatics analyses, the IDP-fusion^4^ tool detects fusion genes by integrating long reads from TGS with short reads from NGS. While this approach offers high accuracy and a very low FP rate, it incurs additional costs due to the need for supplementary NGS sequencing. The PBcR tool, designed for PacBio sequencing, enhances read accuracy to over 99.9% and reduces the proportion of FP chimeric reads to less than 2.5%.^16^ However, these tools are specifically tailored for PacBio sequencing and focus on correcting FP chimeric sequences generated during or prior to library construction. They are unable to address FP chimeric sequences produced during the sequencing process itself. Moreover, there is currently a lack of research that simultaneously characterizes FP chimeric reads arising from both library preparation and sequencing errors on the nanopore sequencing platform. And, no bioinformatics tools are available to filter these FP chimeric reads.

**Figure 1:**
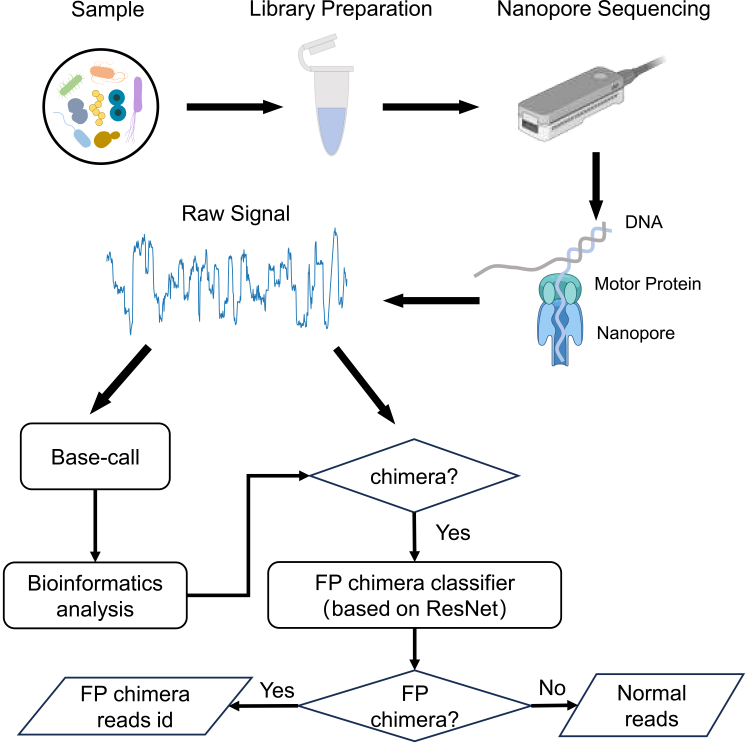
Flowchart of the FP chimeric read filtering process in this study The raw signal data are basecalled and mapped to reference sequences to identify chimeric reads. The raw signal data of these chimeric reads is then processed through a DL-based FP chimera classifier (based on ResNet model), which classifies the reads and outputs the IDs of the FP chimeric reads caused by artifacts.

In this study, we systematically evaluated FP chimeric phenomena in two nanopore sequencing platforms: Oxford Nanopore Technologies (ONT) MinION MK1B^17^ and QitanTech QNome-3841. We compared the electrical signals of FP chimeric reads with those of normal reads and identified a distinguishing signal pattern characterized by a smoothed segment near the breakpoint. Based on these findings, we developed a deep learning (DL) model to classify and filter the FP chimeric sequences from ONT sequencing data (Figure 1).

## Results

### Study design

To identify the FP chimeric reads in nanopore sequencing, we employed three DNA standards (described in the “methods” section) for sequencing on the MinION Mk1B platform. Following bioinformatics analysis, three types of FP chimeric events were identified, with the inverted repeat type being the most prevalent. Since the homemade datasets were all ligation-based, we acquired 45 public datasets of the NA12878 human genome reference standard, prepared using three different library preparation methods, to validate the aforementioned findings and incorporate additional library preparation methods. Consistent with our previous results, three types of FP chimeric events were observed in these datasets, with the FP chimeric rate reaching up to 7.35%.

Subsequently, the electrical signals of FP chimeric reads were visualized, and a representative feature was identified in the corresponding signals. Statistically, this feature was significantly more prominent in FP chimeric reads than in normal ones. Based on these observations, we propose to develop a DL model to classify true and false chimeric events by exploiting the signal differences between them. Finally, the DL model was applied to filter the FP chimeric reads. Somatic SVs were subsequently called, and the error rates were compared between the filtered and raw datasets, confirming that our model effectively reduces the error rates of somatic SV detection.

### The FP chimeric ratio was correlated with read length, library preparation method and basecalling model

**Figure 2:**
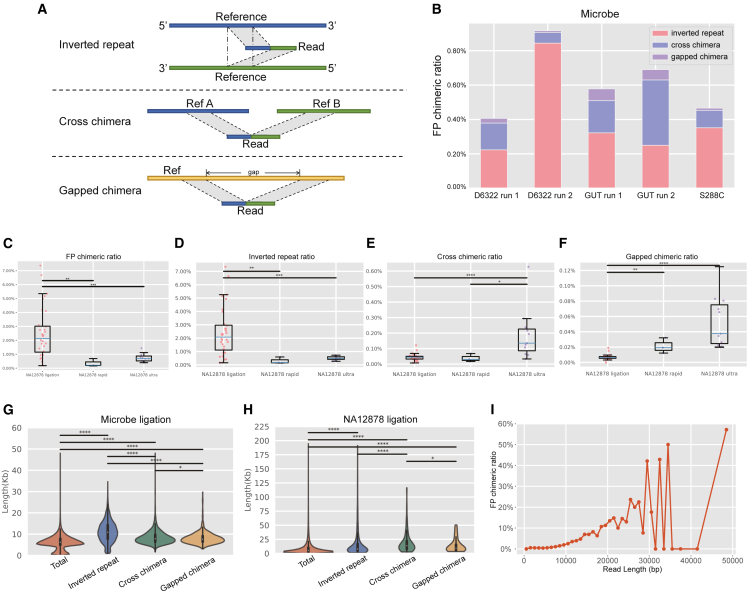
The relationship between FP chimeric ratio and library preparation method and read length (A) Schematic structure of FP chimeras, where different colors represent distinct sub-fragments. (B) FP chimeric ratio of home-made datasets, and the *y* axis is the FP chimeric ratio, defined as the proportion of FP chimeras in all reads. (C–F) Ratios of the three FP chimeric types in NA12878 public datasets generated using ligation (*n* = 31), rapid (*n* = 3), and ultra (*n* = 11) library preparation methods. (G–H) Distribution of read lengths for all reads and FP chimeras in GUT run 1 (G) and NA12878 ligation run 10 (H). (I) Line chart illustrating the relationship between read length and FP chimeric ratio, derived from the GUT run 1 dataset. Data are presented as median and interquartile range unless otherwise specified. Pairwise comparisons in (C–H) were performed using two-sided Mann-Whitney *U* tests. *n* represents independent sequencing runs. No multiple comparison correction was applied. ∗*p* < 0.05; ∗∗*p* < 0.01; ∗∗∗*p* < 0.001.

Three prevalent types of FP chimeras were identified across all datasets: inverted repeat, cross chimera, and gapped chimera (Figure 2A). Detailed information on the three types of FP chimeras across all datasets is provided in Table S1. The average FP chimeric ratio in the five home-made datasets was 0.61% (SD: 0.002) (Figure 2B). The home-made datasets used the ligation method for library preparation, while the public datasets employed three different methods: ligation, rapid, and ultra. Additionally, human and microbiota standards contained distinct genomic and DNA fragments (genome size: 3 Gb vs. < 20 Mb). We subsequently investigated whether read length and library preparation method influence FP chimeric ratios.

Table S1. Basic information of the datasets and the detected chimeric reads

The library preparation methods significantly influenced the FP chimeric ratios in the public datasets (Figures 2C–2F). Datasets prepared using the ligation method exhibited significantly higher total FP chimeric and inverted repeat ratios compared to those prepared with rapid or ultra-methods. The ligation-based datasets demonstrated the highest average FP chimeric ratio (2.26%) among public datasets, with inverted repeat reads showing the highest proportion among the three types of FP chimeras. The maximum FP chimeric ratio reached 7.35%, with inverted repeat structures accounting for 99.73% (7.33%/7.35%). In comparison, the average FP chimeric ratios for rapid-based and ultra-based datasets were significantly lower, measuring 0.17% and 0.69%, respectively (Figures 2C–2F). However, the ratios of gapped chimera and cross-chimera were notably higher in the ultra-library preparation method compared to the ligation method. Additionally, the FP chimeric proportion for the D6322 sample on the QNome-3841 platform was 1.06%, slightly higher than that observed with the MinION Mk1B platform (Table S1).

Subsequently, we evaluated the relationship between read length and FP chimeric ratio. FP chimeras were more likely to occur in longer reads. The length distribution of FP chimeric reads is illustrated in Figures 2G–2J. Furthermore, we divided the chimeras with 1,000 bp intervals and observed a positive correlation between FP chimeric ratio and read length (R = 0.57, *p* = 0.00015) (Figure 2I). This finding is consistent with previous results from the multiple displacement amplification (MDA) method, which demonstrated that the FP chimera rate increases with longer read lengths.^13^ We further investigated the relationship between N50 length and the proportion of FP chimeras across all datasets, revealing no significant correlation based on the Pearson correlation test (Figure S2).

We analyzed the 50 datasets using both the FAST and SUP basecalling models in Guppy to evaluate the impact of different basecalling strategies on the FP chimeric read rate. Unexpectedly, the SUP model exhibited higher average FP chimeric rates than the FAST model across all dataset types (micro: 0.44% vs. 0.39%; NA12878 ligation: 2.19% vs. 0.78%; NA12878 ultra: 0.95% vs. 0.89%; NA12878 rapid: 0.20% vs. 0.15%). The maximum false-positive rates reached 6.15% for the SUP model and 2.25% for the FAST model (Table S2).

Table S2. Chimeric read statistics for the SUP basecalling model and FAST basecalling model

### FP chimeric reads affect somatic SV identification

**Figure 3:**
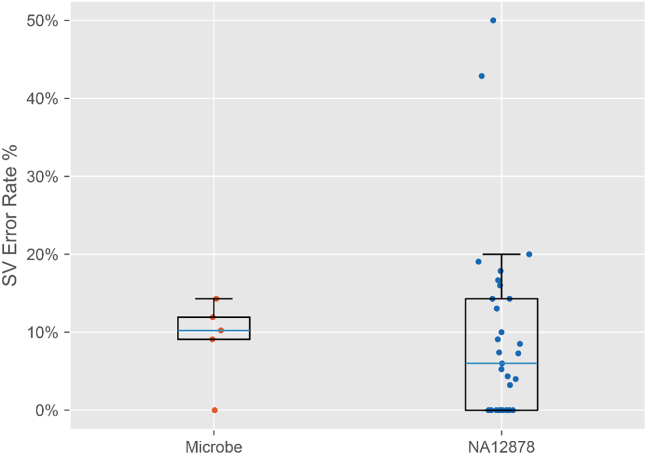
SV error rates caused by FP chimeric reads in ligation-based datasets of NA12878 and microbial standards The SV error rate represents the ratio of false SVs caused by FP chimeric reads to true SVs. NA12878 ligation datasets (*n* = 31) and microbial standard datasets (*n* = 5) are shown. Data are presented as median and interquartile range. *n* represents independent sequencing runs. No statistical comparisons were performed.

To evaluate the impact of FP chimeras on SV detection, we used Sniffles to assess the above 31 public ligation-based datasets and 5 home-made datasets. After filtering the FP chimera, the detected SVs tended to decrease in the clean datasets (Table S3). Thus, FP chimeras may influence somatic SV detection. As shown in Figure 3, our analysis revealed that 66.67% (24/36) of the datasets were affected by FP chimeric reads to varying degrees. Using the previously mentioned method, the average error rate across the 36 datasets was 9.30% (range: 0%–50%, SD: 0.11). In the NA12878 ligation run 16 (error rate: 50%), the clean dataset contained 2 SVs, while the raw dataset contained 1 FP SV, which consisted of a 709 bp deletion. In addition, a 114 bp length insertion SV comprising 84% repeated “GC” in gut run 1 of *Roseburia hominis* was caused by FP chimeras. This homopolymer sequence likely resulted from sequencing error. A 508 bp length insertion SV in NA12878 ligation run 31 was also identified. These three FP SVs were filtered after removal of the FP chimeric reads.

Table S3. Effect of varying --mosaic-af-min values on structural variant detection

### The signal feature of FP chimera

**Figure 4:**
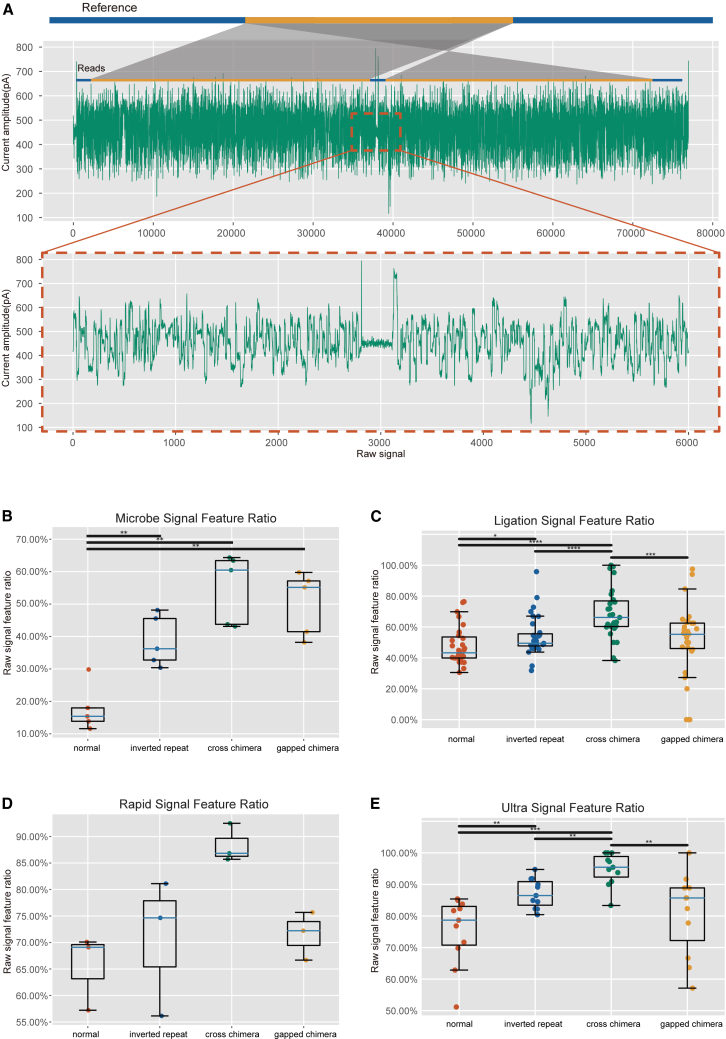
The signal feature of FP chimeric reads (A) The signal characteristics of FP chimeric reads: red dashed lines indicate regions harboring a smoothed segment near the breakpoint. (B–E) Proportion of reads with a smoothed segment near the breakpoint among four types (normal, inverted repeat, cross chimera, and gapped chimera) in microbial datasets (B, *n* = 5) and NA12878 datasets prepared using ligation (C, *n* = 31), rapid (D, *n* = 3), and ultra (E, *n* = 11) library preparation methods. Data are presented as median and interquartile range. Pairwise comparisons among the four types within each panel were performed using two-sided Mann-Whitney *U* tests. *n* represents independent sequencing runs. No multiple comparison correction was applied. Exact *p* values are provided in the figure panels or supplemental tables. ∗*p* < 0.05; ∗∗*p* < 0.01; ∗∗∗*p* < 0.001.

Since nanopore sequencing base-calling relies on electrical signals, we visualized the electrical signals of FP chimeric reads and compared them with normal reads. Notably, we observed a fragment characterized by smoother, lower-amplitude electrical signals embedded within normal amplitude signals, referred to as the “smoothed segment” (Figure 4A). Using Tombo (version 1.5.1), we correlated these signals with nucleotides and discovered the smoothed segment located at the chimeric breakpoints of FP chimeric reads.

To further validate the frequency of the distinguishing feature, electrical signals were obtained from 100 randomly selected reads each from three types of FP chimeric and normal reads (reads without FP chimeric) for manual analysis (Figure S3). The ratio of the smoothed segment among FP chimeras was significantly higher than that in normal reads (47% for inverted repeat reads, 46% for cross-reads, 46% for gapped reads, and 13% for normal reads). To quantify the smoothed segments across all datasets, we implemented a tool in Python (https://github.com/xzhbio/FP-SVs-detector). In every dataset analyzed, the ratio of smoothed segments among FP chimeric reads consistently exceeded that in normal reads. In microbial datasets, FP chimeric reads exhibited a notably higher proportion of smoothed segment compared to normal reads, with 55.02% (SD: 0.106) for cross-chimera, 50.35% (SD: 0.097) for gapped chimera, and 38.61% (SD: 0.078) for inverted repeat harboring such features (Figure 4B). Conversely, the results in the NA12878 dataset were less pronounced (Figures 4C–4E), likely due to variations in experimental conditions and techniques across different laboratories. Nevertheless, the smoothed segments in inverted repeat reads and cross-chimeras still demonstrated a significant difference compared to normal reads. In the rapid library preparation method, no significant differences were observed in the electrical signals between FP chimeras and normal reads. This may be attributed to an insufficient sample size, which can lead to an inability to reveal statistical differences.

We focused on classifying FP inverted repeat reads, as inverted repeats constitute the highest proportion of FP chimeras. Furthermore, inverted repeats are associated with chromosomal fragility and aberrations, potentially leading to chromosomal rearrangements and mutations. These changes may activate oncogenes or inactivate tumor suppressor genes.^18^ Since FP chimeras and true chimeras cannot be classified solely based on sequence, and nanopore sequencing generates raw data in the form of electrical signals, these findings suggest that technologies capable of handling long sequences, such as DL technology, could be employed to effectively distinguish FP chimeras from normal ones. We propose to utilize a DL model based on electrical signals to address this challenge.

### DL-based FP chimera classifier

#### Construction of FP chimera classifier

**Figure 5:**
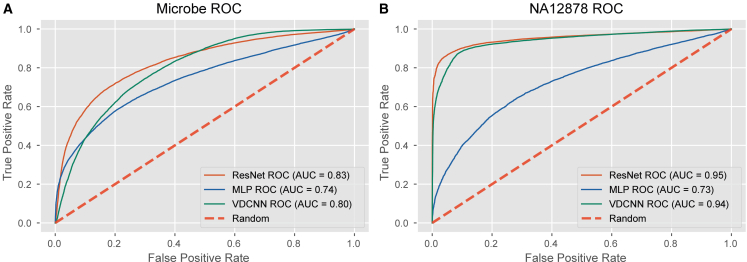
Model evaluation and selection for the microbe and NA12878 datasets (A) The ROC curves of different models in classifying FP chimera in the D6322 run 2 dataset. (B) The ROC curves of different models in classifying FP chimera in the merged datasets composed of NA12878 ligation runs 27–31.

We evaluated the performance of three models—MLP, ResNet, and VDCNN—using the same dataset (D6322 run 1) for training and a separate validation dataset (D6322 run 2). For the NA12878 samples, the ligation runs 1–26 were used for training and runs 27–31 were used for test. ResNet’s performance was slightly better than that of VDCNN and significantly outperformed that of the MLP model. Additionally, ResNet had the largest area under the ROC curve (AUC) than the other models across different datasets (Figure 5). Moreover, ResNet achieved higher accuracy, recall, and other evaluation metrics (Figure S4), further confirming its overall robustness and efficacy. With a batch size of 256 and an epoch of 30, ResNet required 1.4 GB of GPU memory, whereas VDCNN required 2 GB. With respect to runtime, ResNet completed the model training in 58 min, compared to 107 min for VDCNN. Given ResNet’s superior efficiency in both time and computational resources compared to VDCNN as well as its better performance, we selected ResNet as the backbone architecture for the FP chimeric reads classifier.

#### Performance evaluation of FP chimera classifier

**Figure 6:**
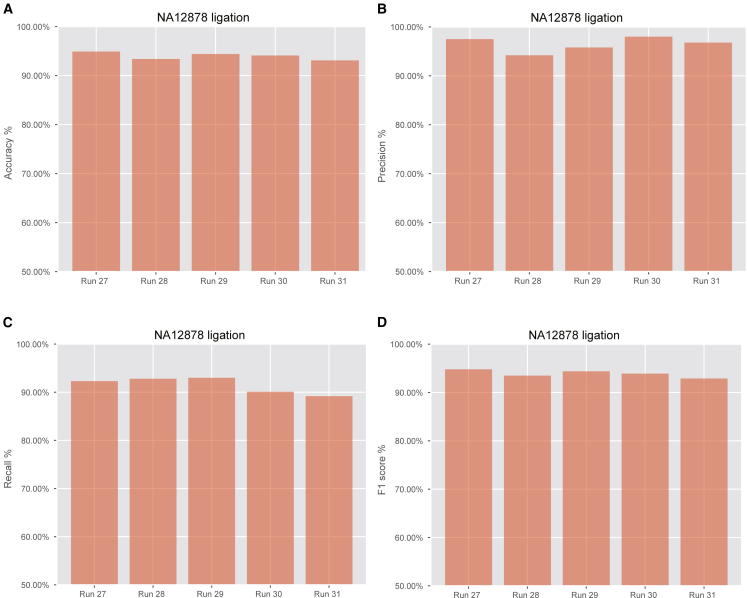
Evaluation of the model on the external NA12878 validation datasets (A) Accuracy of the model on the external NA12878 validation datasets. (B) Precision of the model on the external NA12878 validation datasets. (C) Recall of the model on the external NA12878 validation datasets. (D) F1 score of the model on the external NA12878 validation datasets.

The ResNet-based model trained on the NA12878 dataset, comprising 308,367 read signals, achieved an average accuracy of 93.12% across five external validation datasets, with an average precision of 95.80%, recall of 90.10%, and F1 score of 92.84% (Figures 6A–6D). However, the accuracy of the FP chimera classifier significantly decreased when the model trained on ligation-based datasets was applied to ultra- and rapid-based datasets, yielding accuracies of 61.71% and 74.59%, respectively. The precision of ultra- and rapid-based datasets exceeded 0.95, whereas the recall was below 0.5.

**Figure 7:**
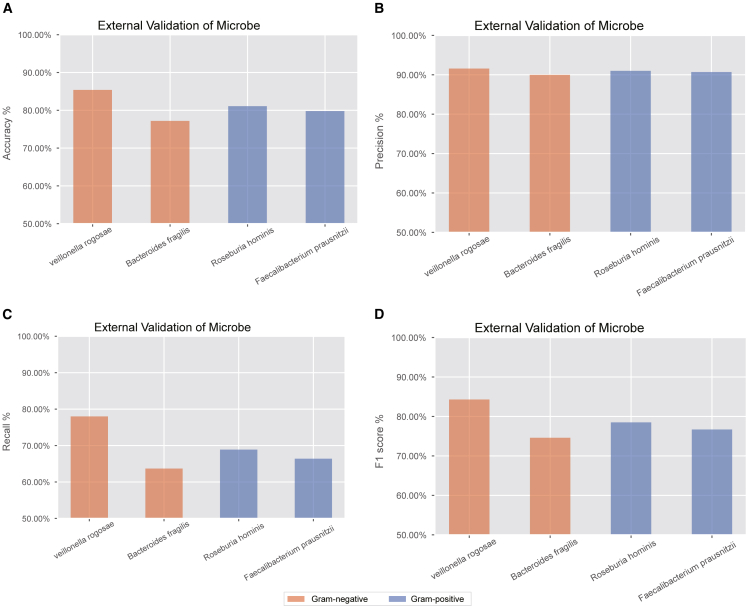
Evaluation of the microbial model’s generalization capability on the four gut species used for validation (A) Accuracy of the model on the four gut species. (B) Precision of the model on the four gut species. (C) Recall of the model on the four gut species. (D) F1 score of the model on the four gut species.

Furthermore, the model trained on the NA12878 datasets exhibited poor performance in the microbial datasets, whereas the model trained on the microbial datasets also underperformed in the NA12878 datasets. To address this, we developed a separate model specifically for microbial data using 64,961 read signals, achieving an average accuracy of 80.88% on external validation datasets, with an average precision of 90.83%, recall of 69.25%, and F1 score of 78.53% (Figures 7A–7D). The model achieved an average accuracy of 81.3% for two Gram-negative bacteria and 80.45% for two Gram-positive bacteria. These results suggest that the classification performance of FP chimeric reads is not significantly influenced by Gram type. Additionally, the model achieved an average accuracy of 88.45% in the microbial test datasets.

#### SV detection on data filtered by FP chimera classifier

**Figure 8:**
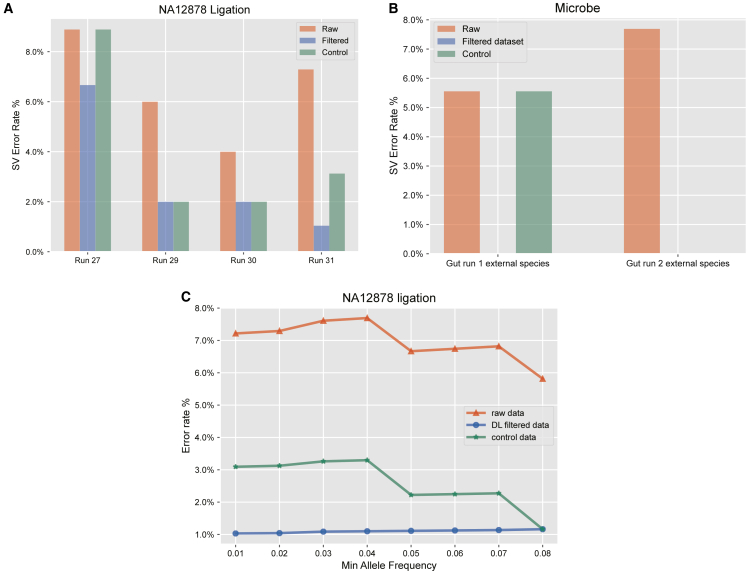
Comparison of the SV error rates among raw datasets and clean datasets filtered by the FP chimera classifier The control data were generated by randomly removing the same number of reads from the raw dataset as were removed in the filtered data. (A and B) Comparison of the error rates of SV detection between raw and filtered data in external validation datasets. (C) Gradient testing of the “--mosaic-af-min” parameter in SV detection across external validation datasets.

To evaluate the performance of the FP chimera classifier, we applied it to filter the raw dataset, thereby removing the FP sequences predicted by the model. Subsequently, we conducted SV detection on the filtered dataset. Control data were generated to incorporate the dose-effect relationship. Following the previously described method, the “--mosaic-af-min” parameter was set to 0.02 to enhance the sensitivity of somatic SV detection, since the proportion of chimeric sequences in gene fusions is minimal. After filtering with the FP chimera classifier, the average error rate for somatic SV detection in the ligation validation datasets reduced from 5.24% (SD: 0.034) to 2.34% (SD: 0.025), and the average error rate for the control dataset was 3.20% (SD: 0.034) (Figure 8A). In the microbial external datasets, the average error rate for somatic SV detection reduced from 6.62% (SD: 0.015) to 0% (SD: 0), and the average error rate for the control data was 2.78% (SD: 0.039) (Figure 8B). In the ultra and rapid datasets, the average error rate for the filtered data reduced by 2.7% and 1.75%, respectively, compared to the raw data (Table S3). These results demonstrate the effectiveness of the FP chimera classifier in filtering FP chimeric reads.

Additionally, we conducted a gradient test on the “--mosaic-af-min” parameter, varying from 0.01 to 0.08, to further validate the FP chimera classifier’s ability to filter FP chimeric reads (Figure 8C). Figure 8 shows a representative dataset from the NA12878 external datasets, while the remaining results are provided in Table S4. Across all gradient ranges, the FP chimera classifier consistently reduced the average error rate by approximately 2%–3%.

Table S4. SV detection for the ligation-related datasets of NA12878 and microbial samples

These results eliminate the potential impact of dose effects and include tests for parameters across all gradient values, demonstrating the efficacy of the FP chimera classifier.

#### Gene fusion detection on data filtered by FP chimera classifier

To evaluate the impact of the FP chimera classifier on the performance of gene fusion detection tools, we tested 31 ligation datasets (fusion detection was performed only on the NA12878 datasets due to the unavailability of annotation files for the microbial samples). No fusion events were identified in four datasets. Among the remaining 27 datasets, 51.9% (14/27) showed a reduction in the number of detected gene fusions to varying degrees after applying the filtering step (Table S5). Among these 14 datasets, between 8% and 100% of the fusion calls (SD: 0.265) were identified as FP gene fusions originating from FP chimeric reads.

Table S5. Effect of the FP chimeric read classifier on NanoFG performance

## Discussion

Gene fusions play a crucial role in cancer diagnosis, making the detection of chimeric sequences essential. The long read generated by TGS technology facilitates the detection of chimeric sequences; however, the FP chimeric reads generated during the sequencing process can significantly impact somatic SV detection results. Furthermore, no method is available to classify and filter artificial chimeric reads produced by nanopore sequencing platforms. Therefore, this study systematically evaluates FP chimeric reads generated by nanopore sequencing. FP chimeric reads were observed to occur in datasets generated by all three library preparation methods, with the highest incidence in the ligation method-related datasets and the lowest in the rapid datasets. In the all 50 public and home-made datasets, neither N50, median, nor average sequencing length is significantly correlated with the FP chimeric ratio. However, the lengths of FP chimeric sequences are significantly greater than those of normal sequences. Additionally, we calculated the FP chimeric ratio for each species within the microbe samples individually and did not observe any discernible pattern. This may suggest that FP chimeric reads occur as random systematic errors.

By visualizing the genomic distribution of FP chimeric reads, we observed that nearly all FP chimeric reads are randomly distributed across the reference genome, which is consistent with the characteristics of sequencing errors, with one notable exception. Specifically, there is a significant enrichment of FP chimeric reads in a specific region. We performed Sanger sequencing on this region and confirmed that the observed changes were due to genuine SVs in the sample.

By visualizing the electrical signal of FP chimeric reads, we identified a smoothed segment flanking the breakpoints, which led to the development of a DL model for classifying FP chimeric reads. We hypothesize that these smoothed segments arise when two consecutive reads translocate through the nanopore too rapidly, causing the sequencer to mistakenly interpret them as a single read. Notably, these segments resemble the smooth signals typically observed at the beginning and end of reads. Using this DL model, we achieved 93.12% accuracy in filtering inverted repeat chimeric reads from the NA12878 datasets and 80.88% accuracy in the microbiota data. However, due to the insufficient data, training models for cross and gapped chimeric reads were not feasible. The datasets filtered by the FP chimera classifier resulted in an average decrease of 2.55% in error rate for somatic SV detection. Although the control group showed decreases in error rates in some datasets, it also led to increased SV detection errors in others. For instance, when the “--mosaic-af-min” parameter was set to 0.01, the error rate increased by 1.4% and 0.77% compared to the raw data in the gut external run 1 and 2 datasets, respectively. For the ultra and rapid datasets, the improvement in error rates through filtering was limited, as the model was not specifically trained for these datasets. Consequently, the ligation-based model exhibited inferior performance on the ultra and rapid datasets.

On the other hand, the FP chimera classifier cannot fully eliminate the SV detection errors induced by FP chimeric reads. Although cross and gapped chimeric reads constitute a small proportion, their impact on SV detection is significant, particularly evident in the ultra-datasets. In the ultra-datasets, the average FP chimeric ratio is only 0.69% (compared to 7.35% in the ligation method), yet the ratio of cross and gapped chimeric reads is approximately three times higher than in the ligation dataset. Consequently, the error rates in SV detection caused by FP chimeric reads in the ultra-datasets are much higher than those in the ligation datasets. It should be noted that our current classifier is specifically trained to identify inverted-repeat chimeric reads. Due to the limited number of gapped and cross chimeric reads in the available datasets, there is currently insufficient data to effectively train models for these types of artifacts. Future work will focus on collecting sufficient examples of FP gapped and FP cross chimeric reads to enable the development of specialized classifiers targeting these rare but impactful artifacts.

In conclusion, the DL model developed in this study demonstrates significant potential for improving the accuracy of chimeric read classification in nanopore sequencing data. By effectively filtering FP chimeric nanopore reads, the model enhances the accuracy of somatic SV detection, reducing error rates by approximately 2%–3% on average, and it also helps eliminate a portion of FP gene fusion calls. This improvement is particularly valuable for cancer diagnosis, where the precise identification of gene fusions and chimeric sequences is critical. However, our tools are currently limited to data generated by the ONT R9 flow cell and chemistry. Future work will focus on extending the analysis to data from the R10 flow cell and chemistry.

### Limitations of the study

This study has several limitations. First, the proposed FP chimera classifier was specifically trained to identify inverted-repeat chimeric reads, as gapped and cross chimeric reads were relatively rare in the available datasets. Consequently, the current model cannot effectively detect these less frequent but potentially impactful chimeric read types, which limits its ability to fully eliminate SV calling errors induced by FP chimeras. Second, the performance of the classifier was optimized for ligation-based library preparation and showed limited generalizability to ultra-long and rapid sequencing datasets, due to the lack of representative training data. Third, although filtering FP chimeric reads reduced somatic SV detection errors, it did not completely remove all FP SV calls, indicating that additional sources of sequencing or alignment artifacts remain. Finally, all analyses were conducted using data generated with the ONT R9 flow cell; therefore, the applicability of the classifier to newer ONT platforms, such as R10, has not yet been evaluated and will require further investigation.

## Resource availability

### Lead contact

Requests for further information and resources should be directed to and will be fulfilled by the lead contact, Ming Ni (niming@bmi.ac.cn).

### Materials availability

This study did not generate new unique reagents or materials. All materials used in this study are commercially available or obtained from publicly accessible resources.

### Data and code availability

• The home-made sequencing data are publicly available from the NCBI BioProjects PRJNA1228318 and PRJNA1083903. All other data reported in the manuscript will be available from the lead contact upon request.
• The FP chimera detection pipeline contains two integrated components: (1) the FP chimera detector module identifies candidate chimeric reads, and (2) the FP chimera classifier processes their corresponding FAST5 files to output the read IDs. The source code is available in the GitHub repository (https://github.com/xzhbio/FP-chimera-classifier).
• Any additional information required to reanalyze the data reported in this paper is available from the lead contact upon request.

## Acknowledgments

This study was supported by the 10.13039/501100001809National Natural Science Foundation of China (grant no. 62472082).

## Author contributions

Conceived and designed the experiments: N.M., Z.J., and X.Z.; performed the experiments: Z.J. and X.Z.; analyzed the data: Z.J., Y.T., and X.Z.; wrote the paper: N.M., Y. Fu, and X.Z.; reviewed and edited the manuscript: Y. Fu and X.Z.; project discussions: N.M., B.X., F.Z., Y. Fu, and Y. Fe. All authors contributed to the article and approved the submitted version.

## Declaration of interests

The authors declare no competing interests.

## STAR★Methods

### Key resources table

**Table:** 

REAGENT or RESOURCE	SOURCE	IDENTIFIER
**Biological samples**		

ZymoBIOMICS™ HMW DNA Standard	ZymoBIOMICS	Catalog No. D6322
ZymoBIOMICS® Gut Microbiome Standard	ZymoBIOMICS	Catalog No. D6331
Saccharomy cescerevisiae ATCC 9763 (S288C)	Testobio	Catalog TQ1000237

**Deposited data**		

Raw data of microbial standard	NCBI BioProjects	PRJNA1228318 and PRJNA1083903
Raw data of NA12878 standard	Github	https://github.com/nanopore-wgs-consortium/NA12878/blob/master/Genome.md.
Original code	Github	https://github.com/xzhbio/FP-chimera-classifier

**Software and algorithms**		

Guppy Version 6.5.7	Oxford Nanopore Technologies	https://nanoporetech.com/
QPreasy Version 1.13.1	QitanTech	https://www.qitantech.com/
Minimap2 Version 2.22	Li et al.^19^	https://github.com/lh3/minimap2
SeqKit Version 2.4.0	Shen et al.^20^	https://github.com/shenwei356/seqkit
Samtools Version 1.16	Danecek et al.^21^	https://github.com/samtools/samtools

### Experimental model and study participant details

#### Microbial DNA standards

Three DNA standards were used to assess the accuracy and reproducibility of sequencing results, enabling a comprehensive analysis of different microbial compositions. The ZymoBIOMICS HMW DNA Standard D6322 (referred to as D6322) and the ZymoBIOMICS Gut Microbiome Standard (referred to as Gut) were purchased from Zymo Research (California, USA) (https://www.zymoresearch.com/). Additionally, the Saccharomyces cerevisiae S288C microbial standard (referred to as S288C) was purchased from Testobio (Zhejiang, China) (https://testobio.com/).

#### Public datasets

To examine the FP chimeric reads generated by different library preparation methods, we utilized FAST5 files from 45 public datasets,^22^ obtained from sequencing the NA12878 human genome reference standard on the MinION Mk1B platform. These datasets were prepared using various library preparation methods: 31 using the ligation preparation method, 3 using the rapid preparation method, and 11 using the modified rapid protocol to produce ultra-long reads (referred to as the “ultra” method). Base-calling for all 45 datasets was performed using Guppy (Version 6.5.7) with the HAC model. The datasets are publicly available via the following link: https://github.com/nanopore-wgs-consortium/NA12878/blob/master/Genome.md.

### Method details

#### Nanopore sequencing with MinION Mk1B

The home-made datasets were sequenced from the aforementioned DNA standards (n=5, including D6322 runs 1 and 2, GUT runs 1 and 2, and S288C) using the MinION Mk1B platform (Oxford Nanopore Technologies, Oxford, UK) and the Ligation Sequencing Kit (SQK-LSK109, Oxford Nanopore Technologies), according to the manufacturer's instructions. In brief, 400 ng of DNA samples was used for end repair and A-tailing with the NEBNext Ultra II End Repair/dA-Tailing Module (New England Biolabs, Massachusetts, USA). Next, adapters were ligated with the NEBNext Quick Ligation Module (New England Biolabs). The 200 fmol library was sequenced on a MinION Mk1B platform with R9.4.1 flow cells (Oxford Nanopore Technologies). Base-calling was conducted using Guppy (Version 6.5.7) with the high accuracy (HAC) model.

#### Nanopore sequencing with QNome-3841

The D6322 standard was sequenced on the QNome-3841 platform (QitanTech, Chengdu, China) using the QLK-V1.1.1 Kit (QitanTech), following the manufacturer’s instruction. In brief, 300 fmol of D6322 DNA was used to end-repair, after which sequencing adapters were ligated. Finally, a 40 fmol library was loaded onto a Qcell-3841 flow cell and sequenced using QNome-3841 platform. Real-time base-calling was performed using QPreasy software (Version 1.13.1, QitanTech) with high-accuracy mode on a desktop workstation (Dell, USA) equipped with a 16 GB NVIDIA A4000 graphics card and an Intel Core i5 Processor.

#### Bioinformatics analysis for chimera detection in nanopore sequencing data

##### Data alignment and preprocessing

FASTQ files were aligned to the reference genome (GRCh38/hg38) using minimap2^19^^,^^23^ (Version 2.22) to produce PAF and SAM files. Reads with MapQ values <60 were excluded to remove secondary alignments and ensure high alignment quality. SeqKit^20^ (Version 2.4.0) was used to calculate Q20, Q30, and N50 metrics for the datasets. Samtools^21^ (Version 1.16) was employed to convert SAM files to BAM format, as well as to sort and index them. Samtools stats provided metrics such as read count, average length, and average alignment quality, while Samtools coverage was used to determine the sample coverage across the reference genome.

#### FP chimeric read detection and classification

Reads that aligned to multiple regions of the reference genome in the PAF files, with at least one supplementary alignment record, were classified as potential FP chimeric reads. These reads were further categorized into three types: inverted repeat, cross chimera, and gapped chimera.

An inverted repeat sequence meets the following criteria: fragments of the reads must align to different strands (positive and negative) on the same chromosome, with each fragment exceeding 500 bp in length. The overlap ratio between the two fragments should be less than 0.05, and their combined length must exceed 80% of the total read length.

A cross chimeric sequence is defined as follows: the read fragments must map to two distinct chromosomes, with each fragment having an alignment length greater than 500 bp. The overlap ratio between the fragments should be less than 0.05, and their combined length must exceed 80% of the total read length. To ensure that the observed chimerism is not a result of homology, the reference chromosome fragments where the two sub-fragments align were extracted and subjected to dual-sequence alignment using BLASTn^24^ (Version 2.14.0+). It is essential to confirm that the sequence similarity is less than 5% by creating a database with one fragment using makeblastdb (Version 2.14.0+) and querying it with the other fragment, evaluating similarity based on the length of the mapped region.

Gapped chimeric sequences involve sub-fragments of the reads that map to two non-contiguous fragments on the same chromosome, with a distance of more than 5000 bp between the reference fragments of the two sub-fragments. Each sub-fragment must have an alignment length exceeding 500 bp, an overlap ratio of less than 0.05, and their combined length must constitute more than 80% of the total read length. The homology check for this classification follows the same procedure as for cross chimeric sequences.

The overlap ratio is calculated using the formula:

(Equation 1) $$\begin{array}{c} overlap-ratio=\frac{overlap\;length}{\min\left(sub-fragment\;map\;length\right)} \end{array}$$

In this context, “overlap length” refers to the number of overlapping bases between two sub-fragments of reads. “min(sub-fragment map length)” denotes the minimum number of bases aligned to the reference sequence for the two sub-fragments.

Based on the above classification criteria, we used the FP chimera detector (https://github.com/xzhbio/FP-chimera-classifier) script to detect potent FP chimeras from the sequencing data.

In this study, we analyzed the genomic distribution patterns of chimeric reads and found that they exhibit no positional enrichment but instead are randomly distributed across the reference genome. Based on this observation, we conclude that all chimeric reads detected in our datasets represent false-positive chimeric reads rather than true chimeric events arising from genuine structural variants.

#### Threshold selection for FP chimera

Using the previously described methods, we classified the FP chimeric reads. Reads with higher overlap-ratio values were more likely attributed to multiple alignments caused by high homology regions in the reference genome, rather than representing true chimeric events. Therefore, we focused on selecting reads with lower overlap-ratio values as candidates for FP chimeras.

By analyzing the overlap-ratio values of FP chimeric reads from the five home-made sequencing runs, we found that over 80% of these FP chimeric reads had overlap-ratio values below 0.2. Based on this observation, the overlap-ratio threshold for FP chimeric reads was set to 0.2, aiming to exclude the influence of multiple alignments and accurately identify true chimeric sequences. The same principle was applied when determining other cut-off values.

#### Implementation of deep learning-based classifier for FP chimeras

##### Model structure

To identify the most effective deep learning-based model for FP chimera classification, we examined the performance of three models capable of handling long sequences: Residual Network^25^ (ResNet), Multi-Layer Perceptron (MLP), and Very Deep Convolutional Neural Network^26^ (VDCNN). Among these models, ResNet achieved the highest accuracy (details provided in the Results Section). The MLP consists of three linear layers, with the following input-output neuron configurations : (20000, 4096), (4096, 64), and (64, 2). The three-dimensional convolutional structure was modified to a one-dimensional convolutional kernel to accommodate the structural characteristics of the electrical signals in the ResNet model. For VDCNN, we modified its first embedding layer to a one-dimensional convolutional layer with a kernel size of 3^27^ to satisfy the characteristics of electric signals.

#### Training, validation and test datasets

The training, validation, and test datasets for the FP chimera classifier were constructed using five home-made microbial datasets and 31 public ligation-based NA12878 datasets, while ultra-based and rapid-based datasets excluded due to insufficient data volume. The home-made datasets were utilized to train the microbe-based model, while 26 of the 31 public ligation-based datasets were used for the human-based model. Electrical signals from all FP chimeric reads, as well as an equal number of normal reads, were randomly selected and divided into three subsets with an 8:1:1 ratio for training, validation, and testing, respectively. Additionally, the electrical signals from two Gram-negative species (*Veillonella rogosae* and *Faecalibacterium prausnitzii*) and two Gram-positive species (*Roseburia hominis* and *Bacteroides fragilis*) in GUT runs 1 and 2 were selected as the external validation dataset for the microbe-based model (referred as GUT external runs 1 and 2). The remaining 5 public ligation-based datasets were used as the external validation dataset for the NA12878-based model to evaluate the model’s generalizability.

#### Data preprocessing

The raw electrical current signals generated by nanopore sequencing exhibit significant length variability, ranging from thousands to hundreds of thousands of signal units. This characteristic impedes the direct application of raw electrical signals to deep learning models. The initial part of a read contains invalid signals, including white noise of nanopore before sequencing. To address this, we trimmed the first 1500 signal points from each read, as most invalid signals contained less than 1500 signal points.^27^ To ensure model input consistency and address the specific features of FP chimeric sequences, we implemented the following strategy: for each chimeric breakpoint, we selected *N* electrical signal points flanking the breakpoint, totaling 2*N* points. For normal reads, we randomly selected 2*N* signal points. Sequences shorter than this length were zero-padded to ensure consistency in data structure. To handle extreme outliers defined as those exceeding three times the average value, we applied a smoothing method that replaced these outliers with the average of neighboring signal points, to mitigate the impact of abnormal fluctuations. Before the padding step, each electrical signal was normalized using Z-score normalization.^28^

To determine the optimal input signal length for the model, a length gradient scan was performed on the preprocessed electrical signals (Figure S1). The model performance was evaluated across different signal lengths, and it was found to reach a plateau at a length of 20,000 (where *N*=10,000). Therefore, 20,000 signal points were selected as the final input length for model training.

#### Structural variation detection

To assess the impact of FP chimeras on somatic SV detection, Sniffles^29^ (Version 2.2) was utilized, which is specifically designed for long-read sequencing technologies such as PacBio and Nanopore sequencing. The “--mosaic” parameter was applied to detect somatic variations. After generating the VCF file with Sniffles, the file was compressed into gz format using BGZIP. Subsequently, the VCF.gz file was indexed using TABIX. Next, FP chimeras were removed from the raw datasets to create clean datasets. Using the method described above, two VCF files were generated from the clean and raw datasets, which were then compared using the VCF-compare command from VCFtools.^30^ The error rate was subsequently calculated to assess the impact of FP chimeras on somatic SV detection. Specifically, the error rate was calculated based on the SV results from the clean datasets, while also accounting for FP and false negatives (FN) in the raw datasets.

#### Gene fusion detection

To assess the influence of our FP chimeric read classifier on gene fusion detection, we employed NanoFG^31^ to identify fusion events using both the unfiltered dataset and the dataset in which false-positive chimeric reads had been removed by our classifier.

### Quantification and statistical analysis

Statistical analyses were performed using Python (version 3.10.8) with the SciPy and scikit-learn libraries. Comparisons between two independent groups were conducted using the two-sided Mann–Whitney U test. Correlations between continuous variables were assessed using Pearson correlation coefficients. Receiver operating characteristic (ROC) curve analysis was performed using scikit-learn, and the area under the curve (AUC) was calculated to evaluate model performance.

Data are presented as median and interquartile range unless otherwise specified. The exact sample size (n) for each experiment is indicated in the corresponding figure legends and represents independent sequencing runs. All statistical tests were two-sided. Exact p values and detailed statistical parameters are provided in the figure legends, Results, or Supplementary Tables, as appropriate. No multiple comparison correction was applied unless otherwise stated.
