# Supplementary material for: Filtering of artificial chimeric reads generated by ligation preparation method of nanopore sequencing
Source: iScience. 2026 Apr 14;29(5):115695. doi: 10.1016/j.isci.2026.115695 (PMC13141652; doi:10.1016/j.isci.2026.115695)
Supplement: Document S1. Figures S1–S4 [file mmc1.pdf]

iScience, Volume *29*

Supplemental information

Filtering of artificial chimeric reads generated by ligation preparation method of nanopore sequencing

Zihan Xie, Jiarong Zhang, Tingting Yang, Xiaochen Bo, Zhiguo Fu, Fengqin Yang, Fuqiang Ye, and Ming Ni

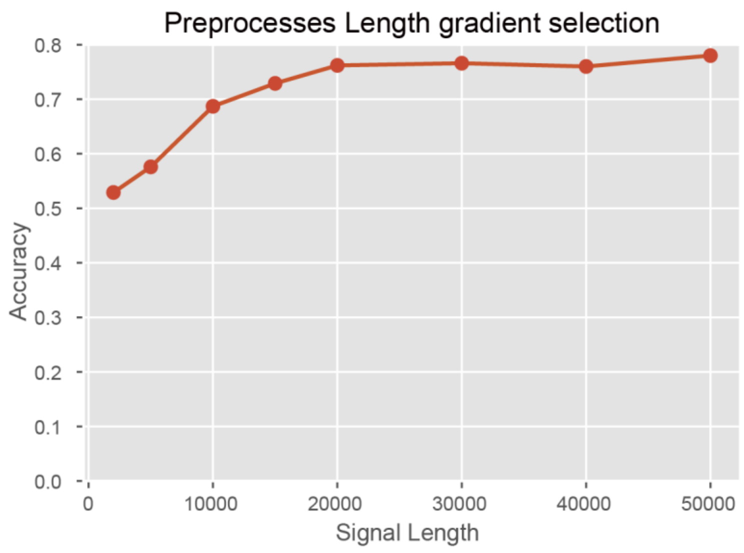

Supplementary Figure 1. Gradient scan of the input signal length for the FP classifier model.

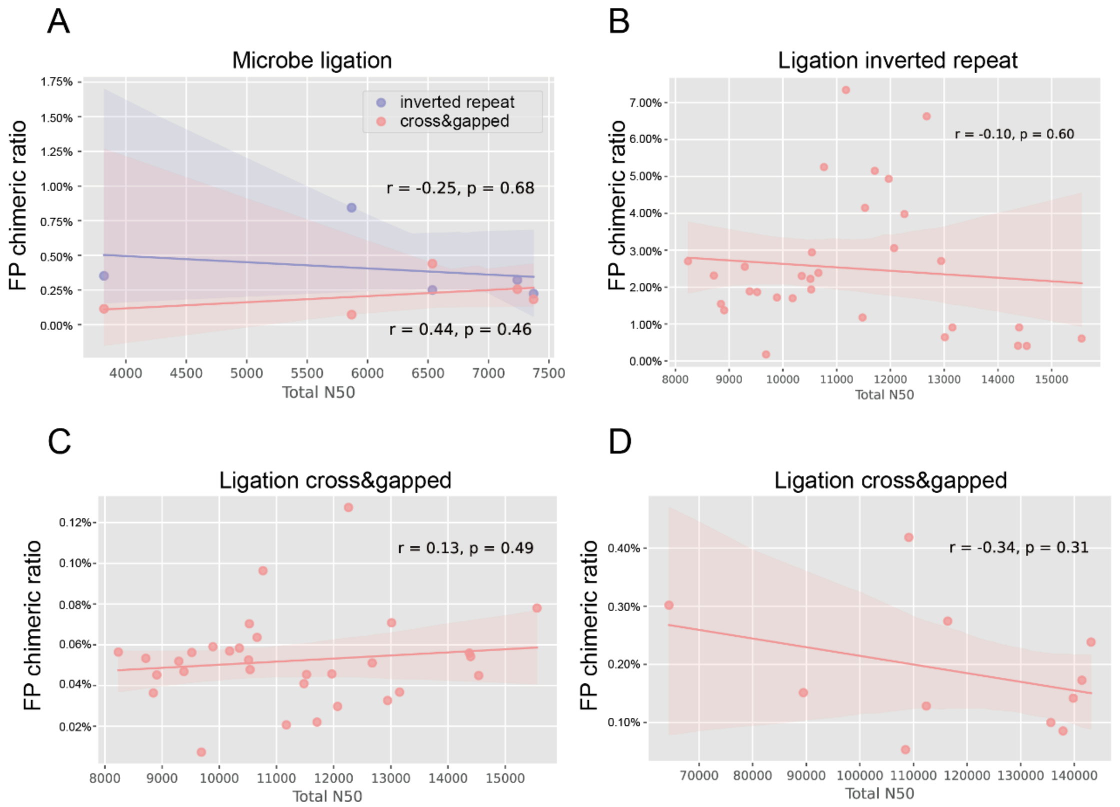

Supplementary Figure 2. Association between sequencing length and proportion of FP chimeric reads.

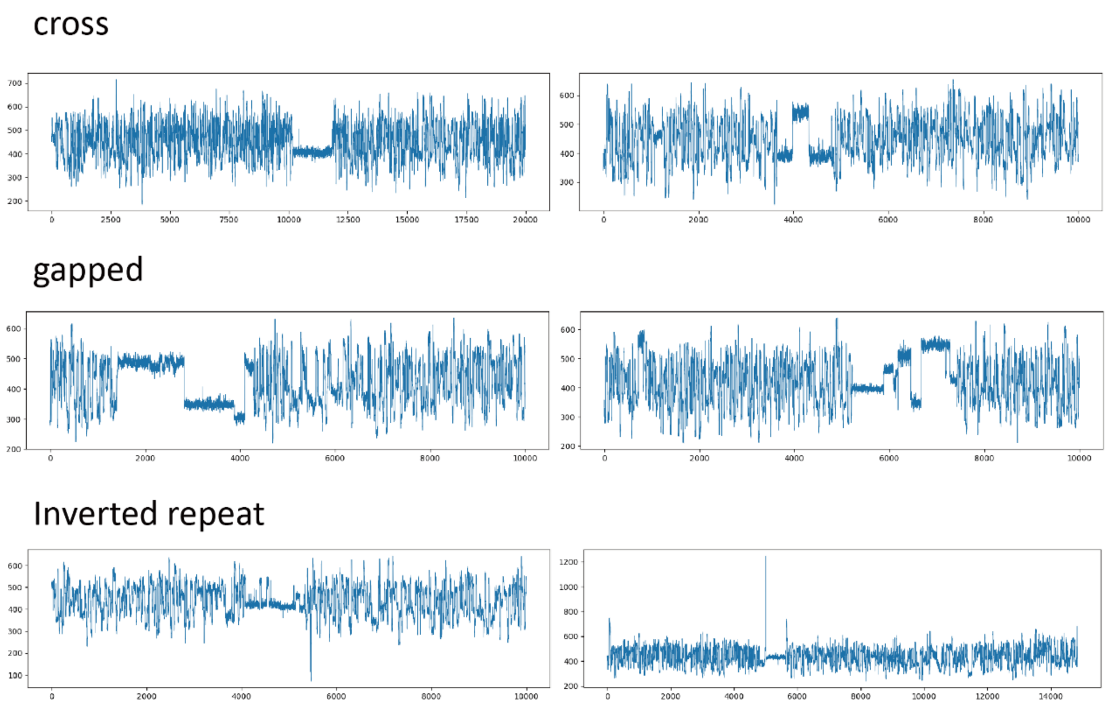

Supplementary Figure 3. The signal feature of cross, gapped and inverted repeat reads.

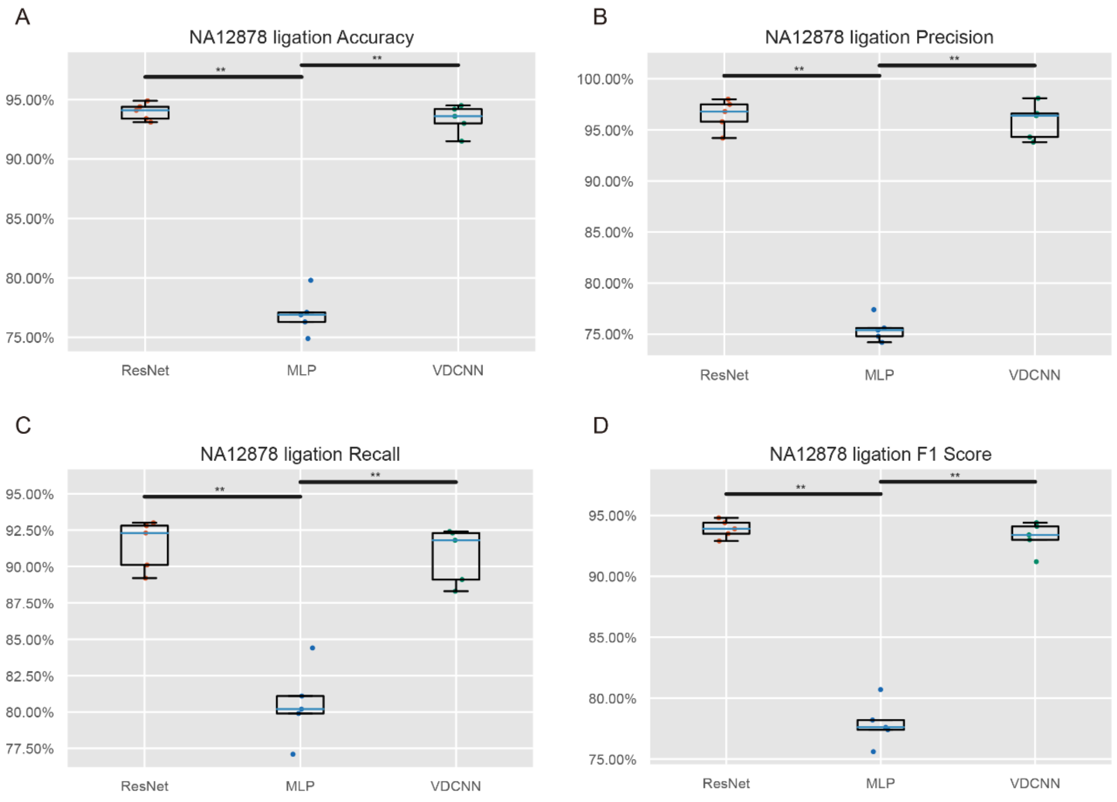

Supplementary Figure 4. Model evaluation and selection for microbe and NA12878.
